# Supplementary material for: A Scoping Review of Galectin-3 as a Biomarker of Cardiovascular Diseases in Pediatric Populations
Source: Int J Environ Res Public Health. 2022 Apr 5;19(7):4349. doi: 10.3390/ijerph19074349 (PMC8998322; doi:10.3390/ijerph19074349)
Supplement: Supplementary file 1 [file ijerph-19-04349-s001.zip › ijerph-1606455-supplementary.pdf]

Supplementary Table S1

**Reference** ***Publication year/*** **[Reference number]**	**Study group (Number of patients) C-children A-adults, Age (R - Range *M- Median*)**	**Control group: Yes/No (Number of individuals) C-children A-adults**	**Study design**	**Heart diseases in study groups**
Mohammed et al. *2014/*[20]	C(60)<br>R:5-36 mo.<br>*M:16 mo.*	Yes<br>C (30)	prospective	ASD<br>VSD<br>AVSD<br>PDA<br>Other congenital heart defects
Numano F et al. *2015*/[24]	(144)<br>C (63)<br>*R: 10-69 mo.*<br>*M: 35,4 mo.*<br>*A (81)*<br>*R: no data*<br>*M: no data*	Yes<br>C (7)<br>A (68)	prospective	Kawasaki Disease
Kotby et al. *2016/*[19]	C(45)<br>R:2 mo–15 y.<br>*M: 9 y.*	Yes<br>C (45)	Prospective, cross-sectional	DCM<br>CHD<br>RHD
Woulfe KC et al. *2017*/[23]	(52)<br>C (42)<br>R:0,05 – 17,7 y.<br>M: 4 y.<br>A(10)<br>R: 20-60 y.<br>M: 51 y.	Yes<br>C (22)<br>A (10)	prospective	IDC
Frank BS et al *2018.*/[28]	C(27)<br>R: 2 d.-12 y.<br>*M: 0,7 y.*	No	Prospective, cohort study	CoA before and after repair.
Zegelbone et al. *2019*/[29]	C(16)<br>R:12-57 y.<br>M:15,5 y.	No	prospective	TOF after repair
Elhewala et al. 2020/[22]	C (40)<br>R: 6-36 mo.<br>M: 15,48 mo.	Yes<br>C(20)	prospective	CHD

Parker et al. *2020*./[25]	C(145)<br>(13 neonates, 52 infants,<br>80 older children)<br><br>*no mortality or readmission group<br>R: 5,2-80,6 mo.<br>M: 34,8 mo.<br><br>*readmission or mortality group<br>R: 4,1-48,7 mo.<br>M: 7,4 mo.	No	Prospective, cohort study	VSD<br>AVSD<br>PVR
Saleh et al. 2020/[21]	C (75)<br><br>R: 0,6-9,3 y.<br>M: 3,7 y.	Yes<br>C (40)	Prospective, cohort study	ASD<br>VSD<br>PDA<br>ASD+VSD<br>PS<br>CMR<br>TGA<br>TOF
Parsons et al. 2020/[27]	C (162)<br>R: 0-18 y.<br>M: 4,05 y.	No	Prospective, observational	CHD
Greenberg et al. 2021/[26]	C(395)<br><br>R: 1 mo. – 18 y.<br>M: no data	No	Prospective, cohort study	CHD
Pietrzak et al. 2021/[31]	C (25)<br><br>R:11-17 y.<br>M: 14,3 y.	C (21)	prospective	Ventricular arrhythmia
Bosch et al. 2021/[30]	C (133)<br><br>R: 10,4-15,9y.<br>M: 13,2 y.	No	Prospective, multicenter	HLHS<br>TA<br>PA<br>DILV<br>DORV
